# Nurses’ Knowledge of Insensible Water Loss and the Evolution of a Digital Decision-Support Tool for Personalized Fluid Balance Assessment: A Cross-Sectional Study

**DOI:** 10.3390/jpm16090456

**Published:** 2026-08-30

**Authors:** Anna Grimaldi, Diego Lopane, Stefano Mancin, Orsola Agorini, Vincenzo Di Nuzzo, Giuseppe Frattolillo, Domenico Di Sivo, Giovanni Gavarro, Incoronata Chiusolo, Ciro Pozzuoli, Sara Morales Palomares, Italian Society of Nephrology Nurses (SIAN) Board of Directors Group, Giovanni Cangelosi, Alessandro Stievano

**Affiliations:** 1 Caserta Local Health Authority—Marcianise Hospital, Viale Sossietta Scialla, 81025 Marcianise, Caserta, Italy; anna.grimaldi@aslcaserta.it (A.G.); vincenzo.diuzzo@aslcaserta.it (V.D.N.); g.frattolillo@aslcaserta.it (G.F.); giovanni.gavarro@aslcaserta.it (G.G.); ciro.pozzuoli@aslcaserta.it (C.P.); 2 IRCCS Humanitas Research Hospital, Via Manzoni 56, 20089 Rozzano, Milan, Italy; diego.lopane@hunimed.eu; 3 Italian Society of Nephrology Nurses, Via Capotesta 1/30, 07026 Olbia, Sassari, Italy; 4 Department of Translational Medical Sciences, University of Campania Luigi Vanvitelli, Via Luciano Armanni 5, 80138 Naples, Italy; orsola.agorini@studenti.unicampania.it; 5 Monaldi Hospital, Azienda Ospedaliera dei Colli, Via Leonardo Bianchi, 80131 Naples, Italy; domenico.disivo@ospedalideicolli.it; 6 Department of Diagnostic and Public Health, University Hospital of Padua, Via Nicolò Giustiniani, 2, 35128 Padua, Italy; incoronata.chiusolo@aopd.veneto.it; 7 Department of Pharmacy, Health and Nutritional Sciences (DFSSN), University of Calabria, 87036 Rende, Cosenza, Italy; 8 School of Pharmacy, Experimental Medicine and “Stefania Scuri” Public Health Unit, University of Camerino, Via Madonna Delle Carceri 9, 62032 Camerino, Macerata, Italy; giovanni01.cangelosi@unicam.it; 9 Department of Clinical and Experimental Medicine, University of Messina, 98100 Messina, Italy; alessandro.stievano@gmail.com

**Keywords:** insensible water loss, clinical decision support systems, personalized medicine, nursing mobile health assessment, cross-sectional study

## Abstract

**Background/Objectives:** Fluid balance management is a core nursing competency, particularly in high-acuity settings. However, the assessment of insensible water loss remains challenging due to the lack of standardized methods, which may increase variability in clinical reasoning. In this context, digital tools may support a more structured and personalized approach. This study aimed to assess nurses’ knowledge of fluid balance and insensible water loss [perspiratio insensibilis (PI)] and to evaluate the usability and perceived quality of a pilot digital clinical decision-support tool (CareBalance-N). Secondary analyses explored differences across clinical settings and the association between knowledge level and app evaluation. **Methods:** A cross-sectional observational study was conducted among 50 nurses working in three clinical settings: hemodialysis (n = 20; 40%), internal medicine (n = 15; 30%), and intensive care (n = 15; 30%). Knowledge was assessed using a structured questionnaire (score range: 0–100), while the usability and perceived quality of the digital application for fluid balance were evaluated using the User Version of the Mobile App Rating Scale (uMARS). Differences between settings were analyzed using ANOVA, and correlations were assessed using Pearson’s correlation coefficient. **Results:** The mean knowledge score was 67.6 ± 7.7, with significant differences across clinical settings [F(2, 47) = 28.4; *p* < 0.001]: hemodialysis 73.6 ± 6.0, intensive care 66.9 ± 4.7, and internal medicine 60.3 ± 5.1. The mean uMARS score was 3.91 ± 0.17, with particularly high ratings for functionality (4.19 ± 0.17) and perceived impact (4.17 ± 0.16). A strong positive correlation was found between knowledge scores and app evaluation (r = 0.943; *p* < 0.001). **Conclusions:** These findings highlight variability in nursing knowledge across clinical settings and suggest that digital tools such as CareBalance-N may represent a promising approach to facilitate more structured clinical reasoning and fluid balance assessment. However, these potential benefits require validation in real-world clinical settings. Further studies are needed to evaluate their impact on decision-making processes, the accuracy of fluid balance assessment, patient safety, and clinical outcomes.

## 1. Background

Fluid balance management is a core component of clinical and nursing practice and is critical to patient safety across a wide range of care settings, including internal medicine, intensive care, and nephrology [1,2]. Accurate assessment of fluid intake and output is essential for guiding clinical decisions and preventing complications such as fluid overload, hypovolemia, and hemodynamic instability, particularly among critically ill patients [3,4]. However, fluid balance should not be regarded as a simple quantitative measure but must be interpreted as a complex clinical process that requires the integration of measurable and non-measurable variables [5]. Among the latter, perspiratio insensibilis (PI) represents a physiological component of daily fluid losses, which occurs through the skin and the respiratory tract and which, by its nature, is not directly quantifiable in clinical practice [6,7]. Despite its pathophysiological relevance, this component is difficult to quantify and is often estimated inconsistently, contributing to substantial variability in fluid balance assessment among professionals and care settings [8]. A major unresolved issue is the absence of a single and universally accepted formula for the estimation of PI, whose quantification is based on empirical and non-standardized models [9,10]. The strategies currently used are based on empirical and simplified approaches, such as estimation based on body weight (for example 10 mL/kg/24 h), possibly corrected in the presence of clinical factors such as fever, mechanical ventilation or increased respiratory work [11]. However, such models are intrinsically limited, as they are not able to capture the physiological complexity and the interindividual variability of insensible losses. This lack of standardization contributes to making fluid balance a real “grey zone” of nursing clinical reasoning, characterized by margins of uncertainty and interpretative subjectivity [12,13]. The relevance of this issue increases in high-complexity care settings, in which even modest discrepancies in the evaluation of hydration status can determine significant clinical consequences [9,10,11,12,13]. In nephrology and intensive care, for example, an inaccurate estimation of fluid losses can influence ultrafiltration strategies, fluid management and the overall hemodynamic balance of the patient [14,15,16]. Furthermore, variability in the consideration of PI can compromise continuity of care, especially in patients transitioning between different clinical settings, where different assessment approaches may coexist [17,18]. In this scenario, the paradigm of personalized medicine (PM) offers a theoretical and operational approach capable of overcoming the limits of traditional standardized models [19]. PM is based on the adaptation of clinical decisions to the specific characteristics of the individual patient, integrating physiological variables, clinical conditions and contextual factors [20,21]. In the context of fluid management, this implies the need for tools and models capable of supporting a dynamic and individualized evaluation of fluid balance, particularly in critical care [22]. Digital technologies and clinical decision support systems currently represent one of the main tools for the implementation of PM in clinical practice [23,24]. In particular, mobile applications and digital tools allow the integration of a multiplicity of clinical and physiological variables into structured decision-making models, improving the quality of clinical reasoning and reducing inter-operator variability. These tools are intended to support augmented intelligence rather than replace clinical judgment [23,24,25]. In the nursing context, the adoption of digital decision support tools plays a strategic role, as it can promote greater standardization of care processes and improve the accuracy of clinical assessment [23,24]. Despite the growing development of digital solutions in healthcare, the literature still highlights a lack of tools specifically designed to integrate PI into nursing fluid balance, thus revealing an important knowledge and application gap [26]. This gap appears particularly critical in a field characterized by high decision-making complexity and significant inter-operator variability [27]. In this context, the development of digital decision support tools represents a promising strategy to improve the evaluation of fluid balance and to support a more structured and individualized approach to clinical practice [28,29,30]. Such tools allow the systematic integration of clinical, physiological and contextual variables, overcoming the limits of traditional approaches based on empirical estimates and simplified models [27,28,29,30,31]. In particular, the possibility of making decision-making processes explicit and traceable can contribute to reducing inter-operator variability and improving the consistency of clinical evaluation, especially in areas characterized by high uncertainty, such as the management of insensible losses [1,2,3,4,5]. In this perspective, digital technologies act as key tools for the implementation of PM, promoting the adaptation of clinical decisions to the specific characteristics of the patient through a dynamic and multidimensional assessment [32,33,34].

The primary aim of the present study was to assess nurses’ knowledge of fluid balance and PI and to evaluate the usability and perceived quality of a pilot digital clinical decision-support tool (CareBalance-N) from a PM perspective. Secondary exploratory aims included comparing knowledge levels across different clinical settings and exploring the association between nurses’ knowledge and app evaluation.

## 2. Materials and Methods

### 2.1. Study Design

This study was designed as a descriptive cross-sectional observational study. The study was reported in accordance with the STROBE (Strengthening the Reporting of Observational Studies in Epidemiology) guidelines for observational studies (Supplementary File S1) [35].

### 2.2. Setting and Recruitment

Data collection was conducted in three different care settings characterized by different levels of clinical complexity and involvement in fluid balance management: hemodialysis units, internal medicine wards, and intensive care units in two Italian tertiary hospitals. The selection of these settings was based on the relevance of fluid balance management within the different care contexts and on the opportunity to include different clinical approaches and levels of professional experience across the continuum of care. Nurses working in the selected settings and meeting the eligibility criteria were invited to participate through non-probability convenience sampling. Inclusion criteria were a minimum of three months of work experience in the relevant clinical setting and willingness to participate voluntarily in the study. The final sample size was determined by the number of eligible nurses available in the involved settings and by voluntary participation during the data collection period. Given the exploratory nature of the study, no formal sample size calculation or statistical power analysis was performed. Questionnaires with more than 20% missing data were excluded from the analysis. Participation was anonymous and voluntary.

### 2.3. Tools

The CareBalance-N application was developed as a digital prototype to support nursing clinical reasoning in fluid balance management. The tool integrates a structured interface for the entry of patient-specific parameters and a calculator for measurable fluid balance components, including ultrafiltration volumes. It also provides an indicative estimate of PI based on a reference value of 10 mL/kg/24 h derived from clinical recommendations [10]. This baseline estimate is contextualized using the clinical and environmental variables included in the prototype, namely body temperature, respiratory conditions, mechanical ventilation, and environmental factors. The application presents the result as an indicative patient-specific value or range. The present study did not evaluate the accuracy of these estimates or the separate contribution of the clinical and environmental variables. The output should therefore be interpreted as support for clinical reasoning rather than as a prescriptive recommendation. CareBalance-N is intended to integrate patient-specific information while maintaining the central role of healthcare professionals in decision-making.

### 2.4. Study Procedure

For the purposes of the study, the CareBalance-N tool was introduced to participants through a dedicated 120 min training session. After 72 h, participants were presented with three simulated clinical cases reflecting the care settings included in the study. Data were collected using two main tools, completed within 24 h after participants had worked through the simulated clinical cases. Considering that the assessment was performed after the standardized training session and the interaction with the CareBalance-N application through simulated clinical cases, the knowledge questionnaire was intended to assess participants’ knowledge at the time of evaluation rather than baseline pre-existing knowledge. The first tool was a structured questionnaire developed ad hoc to assess nursing knowledge on fluid balance and PI. It was developed a priori by a panel of five expert nephrology nurses, based on the main knowledge domains considered relevant for fluid balance management and PI prevention, integrating both theoretical concepts and clinically oriented scenarios. The items were developed to reflect the educational content addressed during the training session and were subsequently reviewed by the expert panel to ensure relevance, clarity, and consistency with the objectives of the study. Correct answers were defined a priori by the expert panel based on current evidence and established clinical principles related to fluid balance management and PI prevention. The questionnaire consisted of 25 items, including theoretical questions and clinical vignettes, with dichotomous scoring (1 point for correct answers, 0 for incorrect or “I don’t know”), and scores were subsequently converted to a 0–100 scale. The questionnaire was developed specifically for the exploratory purposes of the present study. No formal pilot testing was conducted before its application, and no psychometric analyses, including assessment of internal consistency, were performed, as the tool was not intended as a standardized measurement instrument. The second tool was the Italian version of the User Version of the Mobile App Rating Scale (uMARS) [36], to evaluate usability and perceived quality of the application. The scale includes 23 items across six domains (engagement, functionality, aesthetics, information quality, subjective quality, and perceived impact), rated on a 5-point Likert scale. The overall score was calculated as the mean of domain scores. The primary outcomes were the nursing knowledge score (0–100 scale) and the overall uMARS score (1–5 scale). Secondary outcomes included the scores of individual uMARS domains, differences across care settings, and the association between knowledge level and app evaluation.

### 2.5. Statistical Analysis

Statistical analysis was conducted using Jamovi software (Version 2.6, 2024) [37]. Categorical variables were described using absolute frequencies and percentages, while continuous variables were reported as mean and standard deviation or median and interquartile range (IQR), depending on data distribution. Normality was assessed using the Shapiro–Wilk test and graphical inspection (histograms and Q–Q plots). Comparisons across care settings (hemodialysis, internal medicine, and intensive care) were performed for nursing knowledge scores using one-way ANOVA for normally distributed variables, after checking homogeneity of variances with Levene’s test. Post hoc Tukey tests were conducted when significant differences were found. For non-normally distributed data, the Kruskal–Wallis test was used. Effect size for one-way ANOVA was calculated using eta squared (η^2^), interpreted according to Cohen’s (1988) benchmarks (small: 0.01; medium: 0.06; large: ≥0.14) [38]. The r index was used for non-parametric tests. The evaluation of the CareBalance-N application using the uMARS scale was primarily analyzed through descriptive statistics to characterize perceived usability, quality, and acceptability of the prototype. The stratification of uMARS scores according to care setting was performed as an exploratory descriptive analysis aimed at identifying potential patterns of variation across different clinical contexts. Since the study was designed as a preliminary observational evaluation of a digital prototype rather than as a comparative study of usability outcomes between care settings, inferential statistical testing of differences in uMARS scores between groups was not planned. Associations between nursing knowledge scores, uMARS scores, and main socio-professional variables were assessed using Pearson or Spearman correlation coefficients, depending on data distribution. Statistical significance was set at *p* < 0.05.

### 2.6. Ethical Considerations

This study involved only the collection of anonymous data from healthcare professionals, without involving patients or sensitive clinical data. Participation was voluntary and based on informed consent for the processing of personal data for research purposes.

The study was conducted in accordance with the ethical principles of the Declaration of Helsinki [39] and in compliance with Regulation (EU) 2016/679, the General Data Protection Regulation (GDPR) [40]. The study protocol was reviewed and approved by the Scientific Committee of the Italian Society of Nephrology Nurses on 9 February 2026 (protocol code: SIAN-RIC.03/26). The participating institutions considered this approval sufficient and did not require an additional local ethics review because the study was non-interventional, involved no patients, and participation by healthcare professionals was voluntary and anonymous.

## 3. Results

### 3.1. Sample

The study included a total of 50 nurses distributed across three care settings: hemodialysis units (n = 20; 40.0%), internal medicine wards (n = 15; 30.0%), and intensive care units (n = 15; 30.0%). The sample showed moderate heterogeneity in socio-professional variables. The mean age was 40.0 ± 6.9 years, and the mean total work experience was 16.0 ± 6.9 years. Experience within the current clinical setting averaged 7.0 ± 3.2 years. Most participants (64.0%) reported having received specific training in nephrology. The socio-demographic and professional characteristics of the sample are reported in Table 1.

### 3.2. Nursing Knowledge

The overall mean nursing knowledge score on fluid balance and PI was 67.6 ± 7.7, with values ranging from 52 to 84. The distribution showed moderate variability, with a median of 68 (IQR 60–72). Stratified analysis by care setting showed differences in observed knowledge scores. Nurses working in hemodialysis units reported the highest mean scores (73.6 ± 6.0; median 72; IQR 68–80; range 64–84), followed by those in intensive care (66.9 ± 4.7; median 68; IQR 64–72; range 60–72). The lowest scores were observed in internal medicine wards (60.3 ± 5.1; median 60; IQR 56–64; range 52–68). Overall, a progressive difference in observed knowledge scores was identified across settings, with the highest values observed among hemodialysis nurses and the lowest among internal medicine nurses (Table 2). Inferential analysis using one-way ANOVA confirmed statistically significant differences between groups [F(2, 47) = 28.4; *p* < 0.001; η^2^ = 0.55], indicating a large effect size. Post hoc analyses (Tukey test) showed significant differences across all settings, with higher scores observed in hemodialysis compared to internal medicine (Δ = +13.3; *p* < 0.001) and intensive care (Δ = +6.7; *p* = 0.004), as well as higher scores in intensive care compared to internal medicine (Δ = +6.6; *p* = 0.012).

### 3.3. Usability and Perceived Quality of the Tool

The overall mean uMARS score was 3.91 ± 0.17, indicating a good level of perceived quality and usability of the tool. Descriptive analysis of individual domains showed generally high mean values, with the highest scores observed in functionality (4.19 ± 0.17) and perceived impact (4.17 ± 0.16), followed by information quality (4.08 ± 0.16). Lower, although still positive, scores were observed for engagement (3.70 ± 0.18), aesthetics (3.59 ± 0.17), and subjective quality (3.69 ± 0.18). Stratified descriptive analysis by care setting showed some variation in mean uMARS scores, with values of 4.02 ± 0.16 in the hemodialysis group, 3.90 ± 0.12 in intensive care, and 3.78 ± 0.12 in internal medicine. These differences were descriptively reported; however, no inferential statistical comparison between clinical settings was performed, as the study was designed as a preliminary exploratory evaluation of the tool rather than a comparative assessment of usability outcomes across groups. Overall, the findings indicate positive perceived usability and acceptability of the tool across the evaluated clinical settings (Table 3).

### 3.4. Correlation Analysis Between Nursing Knowledge and Digital Tool Evaluation

Pearson correlation analysis showed a strong positive and statistically significant association between nursing knowledge scores and overall uMARS scores (r = 0.943; *p* < 0.001). Weak positive correlations were also observed between knowledge scores and selected socio-professional variables, including age (r = 0.288; *p* = 0.043), total work experience (r = 0.288; *p* = 0.043), and experience in the current care setting (r = 0.291; *p* = 0.041). Although higher knowledge scores were associated with more favorable CareBalance-N evaluations in this sample, both measures showed parallel descriptive gradients across clinical settings, with the highest values in hemodialysis, intermediate values in intensive care, and the lowest values in internal medicine. The unadjusted correlation may therefore partly reflect between-setting differences. No adjusted or partial analysis was performed, and the setting-specific groups were small (n = 15–20); therefore, the coefficient should not be interpreted as an association independent of clinical setting (Table 4).

## 4. Discussion

This study aimed to assess nursing knowledge regarding fluid balance management, with particular focus on PI, and to explore the contribution of a digital decision-support tool within this clinical context. Overall, the findings highlight differences in observed knowledge scores across care settings and provide an exploratory evaluation of CareBalance-N as a support tool for nursing clinical reasoning. These findings are consistent with the literature, which identifies hydration assessment as a complex clinical process characterized by high inter-operator variability and strong dependence on clinical interpretation [41]. In particular, previous research emphasizes that fluid balance should not be considered a purely quantitative parameter but rather requires a dynamic and integrated approach [42,43], supporting the findings of the present study. PI represents one of the most critical elements of this process and its quantification is based on empirical and non-standardized estimates, making it difficult to integrate into clinical decision-making models [44]. This issue has also been highlighted in critical care research, which has questioned the reliability of fluid balance as a clinical indicator, noting that non-measurable variables may compromise its accuracy [45]. The findings of this study align with this perspective, showing that such complexity is reflected in nursing knowledge levels. Differences observed across care settings are consistent with evidence from hemodialysis, internal medicine, and critical care contexts, where fluid management requires a highly individualized approach based on continuous reassessment and the integration of multiple parameters [46]. The present findings extend this evidence by specifically focusing on nurses’ knowledge related to fluid balance management and the estimation of PI losses, an aspect that remains largely dependent on clinical judgment and empirical approaches [2,12].

In less specialized settings, lower exposure to such complexity may result in greater variability in decision-making approaches. These findings are consistent with theoretical models of PM, which emphasize that decision-making quality depends on the ability to dynamically integrate clinical, biological, and contextual variables [47]. However, the literature also highlights that the practical implementation of these models is often limited by difficulties in systematically integrating complex and non-standardized variables, such as insensible losses [48]. The observed variability in knowledge underscores the need to strengthen nursing education on fluid balance, particularly in less specialized settings. Evidence suggests that fluid management requires advanced and up-to-date competencies to support complex, individualized decision-making processes [49]. Targeted educational interventions may help address knowledge gaps and promote more consistent approaches to fluid balance management. Digital decision-support tools may also have a role in this context. Reviews have suggested that clinical decision support systems may contribute to more structured clinical processes and promote standardization while preserving adaptability to individual patient conditions [50,51,52]. Digital tools may support ‘adaptive standardization’ models, balancing more consistent decision-making with adaptability to individual clinical conditions, an approach particularly relevant to fluid balance management given the complexity of the variables involved [53]. From an organizational perspective, shared digital tools may facilitate greater consistency and make decision-making criteria more explicit across professionals and care settings [54,55]. The appropriate use of decision-support tools for PI estimation also depends on the competence of healthcare professionals. The literature indicates that estimating insensible losses requires advanced interpretive skills, as it relies on non-measurable variables and empirical models [56]. Therefore, effective adoption of digital tools requires not only technological availability but also adequate training and critical use, ensuring proper integration into clinical decision-making processes [57]. Future research should prospectively evaluate digital decision-support tools for PI estimation in real-world clinical settings, including their impact on fluid balance accuracy, decision-making processes, patient safety, and clinical outcomes [58,59].

### 4.1. Limitations

In light of the descriptive cross-sectional observational design adopted, the findings of this study should be interpreted with caution, as they do not allow for the establishment of causal relationships but only for the identification of associations among the variables considered. While this methodological choice is consistent with the exploratory nature of the study, it limits the ability to assess the long-term effectiveness of the CareBalance-N application and its impact within real-world clinical workflows. A further critical aspect concerns the use of non-probability convenience sampling and the relatively small sample size (n = 50), which was determined by the number of eligible nurses available in the participating settings and by voluntary participation, without a formal sample size calculation or statistical power analysis. Although quota distribution across care settings was adopted to ensure representation of different clinical contexts along the continuum of care, the sample characteristics limit the generalizability of the findings and their transferability to other populations or healthcare settings. From a tool-related perspective, the CareBalance-N application represents a digital prototype still under development, introduced through a standardized training session and evaluated within a simulated environment using ad hoc clinical cases. Since all participants received the same educational intervention before assessment and no baseline knowledge evaluation or comparison group was included, it was not possible to distinguish pre-existing knowledge levels from knowledge potentially acquired during the training session. Therefore, the observed knowledge scores should be interpreted as reflecting participants’ knowledge at the time of evaluation rather than as a direct measure of educational effectiveness. Furthermore, the simulated nature of the evaluation does not allow definitive conclusions regarding the integration of the application into routine clinical practice or its potential effects on patient outcomes, safety, and decision-making processes in real-world conditions. A further consideration concerns the estimation model implemented within the application. The PI estimate begins with a reference value derived from the literature and is contextualized using clinical and environmental variables. However, this study did not evaluate the accuracy of the resulting estimates or the separate contribution of those variables. The model also does not fully capture physiological complexity or individual variability. Accordingly, the application output should be interpreted as an indicative support element rather than as a prescriptive recommendation. The measurement instruments also present relevant limitations. The nursing knowledge questionnaire, although developed by an expert panel and based on clinically relevant domains, was specifically designed for the exploratory purposes of this study and did not undergo formal psychometric validation or assessment of internal consistency. Similarly, uMARS evaluates perceived usability, quality, and acceptability of digital applications through self-reported measures and primarily reflects subjective user perceptions rather than objective performance indicators. In this context, the very strong correlation observed between nursing knowledge scores and uMARS ratings should be interpreted cautiously. Both measures were collected from the same participants within a limited interval after interaction with the application; common method bias and response tendencies may therefore have contributed to its magnitude. More importantly, both measures followed the same descriptive gradient across clinical settings, raising the possibility that the unadjusted correlation partly reflects between-setting differences. No adjusted or partial analysis was performed, and the setting-specific subgroups were small (n = 15–20); therefore, the present data do not establish whether the association persists within settings or independently of clinical setting. The reported coefficient should therefore be regarded as an exploratory, unadjusted aggregate association influenced by individual and contextual factors, rather than as evidence of an independent or causal relationship. These considerations highlight the need for further studies aimed at strengthening the validity of measurement tools and integrating objective indicators of performance and clinical outcomes. Overall, these limitations point to the need for future research using longitudinal and experimental designs, with larger and more representative samples, conducted in real clinical settings, in order to more rigorously assess the effectiveness, validity, and practical impact of the proposed tool.

### 4.2. Implications for PM

The findings of this study are consistent with the principles of PM, in which fluid balance management—particularly the estimation of insensible losses—represents a clinically complex domain characterized by significant interindividual variability [60]. Unlike standardized approaches based on average reference values, clinical practice requires continuous recalibration of decisions based on the dynamic interaction between physiological variables (e.g., hemodynamic status, respiratory function, body temperature), clinical conditions (such as mechanical ventilation and comorbidities), and environmental factors [61]. In this context, PM emphasizes an adaptive and iterative process of data interpretation over time rather than relying solely on static risk stratification [62]. Within this framework, digital clinical decision support tools such as CareBalance-N may contribute to making clinical reasoning processes more explicit, traceable, and reproducible, facilitating the structured integration of heterogeneous data into context-sensitive estimates [63]. In particular, the possibility of modulating the estimation of insensible losses through the inclusion of modifying clinical variables may represent an initial step toward a more data-informed and individualized approach, potentially helping to overcome some limitations of rigid empirical methods and reducing unwarranted variability across professionals and care settings [64]. Nevertheless, the implications of the present findings for PM should be interpreted with caution, as the current study evaluated knowledge acquisition and usability rather than clinical effectiveness or patient outcomes. The broader contribution of tools such as CareBalance-N to PM will depend on their evolution toward more advanced models of data integration, incorporating longitudinal information, temporal trends, and, prospectively, continuous monitoring data [65,66,67,68,69,70,71]. These developments may support more personalized and dynamic fluid management; however, prospective studies conducted in real-world clinical settings are needed to determine their actual impact on clinical decision-making and patient outcomes.

## 5. Conclusions

This study highlights significant variability in nursing knowledge regarding fluid balance management, particularly in the estimation of PI, confirming the difficulty of translating physiological phenomena that are not directly measurable into structured decision-making processes. This heterogeneity reflects the limitations of empirical approaches and suggests the need for more integrated models. PM may represent a potentially useful interpretative framework based on the dynamic integration of clinical and contextual variables; however, its application remains limited by the lack of standardization of some key components. Digital decision support systems, such as CareBalance-N, may represent a promising approach to facilitate more structured clinical reasoning and fluid balance assessment, particularly in the estimation of PI. However, based on the findings of the present study, these benefits should be regarded as potential and require validation in real-world clinical settings. Such tools should therefore complement, rather than replace, clinical judgment. Future studies conducted in real-world clinical practice are needed to evaluate their impact on decision-making processes, workflow integration, the accuracy of fluid balance assessment, patient safety, and clinical outcomes.

## Figures and Tables

**Table 1:** Sample characteristics.

Variable	Total (n = 50)
Age (years), mean ± SD	40.0 ± 6.9
Total work experience (years), mean ± SD	16.0 ± 6.9
Experience in current setting (years), mean ± SD	7.0 ± 3.2
Care setting, n (%)	
Hemodialysis	20 (40.0)
Internal Medicine	15 (30.0)
Intensive Care	15 (30.0)
Specific training in nephrology, n (%)	
Yes	32 (64.0)
No	18 (36.0)

Legend. SD: Standard Deviation; n: number.

**Table 2:** Overall nursing knowledge score and by care setting.

Care Setting	n	Mean ± SD	Median	IQR	Min–Max
Hemodialysis	20	73.6 ± 6.0	72	68–80	64–84
Internal Medicine	15	60.3 ± 5.1	60	56–64	52–68
Intensive Care	15	66.9 ± 4.7	68	64–72	60–72
Total	50	67.6 ± 7.7	68	60–72	52–84

Legend. SD: Standard Deviation; n: number; IQR: Interquartile Range.

**Table 3:** Evaluation of the CareBalance-N application using uMARS: overall score and domains, total and by care setting.

Domain	Total (n = 50)	Hemodialysis (n = 20)	Internal Medicine (n = 15)	Intensive Care (n = 15)
Engagement, mean ± SD	3.70 ± 0.18	3.84 ± 0.18	3.55 ± 0.11	3.67 ± 0.12
Functionality, mean ± SD	4.19 ± 0.17	4.30 ± 0.17	4.05 ± 0.11	4.17 ± 0.12
Aesthetics, mean ± SD	3.59 ± 0.17	3.71 ± 0.15	3.45 ± 0.11	3.57 ± 0.12
Information quality, mean ± SD	4.08 ± 0.16	4.17 ± 0.17	3.95 ± 0.11	4.07 ± 0.12
Subjective quality, mean ± SD	3.69 ± 0.18	3.81 ± 0.17	3.55 ± 0.11	3.67 ± 0.12
Perceived impact, mean ± SD	4.17 ± 0.16	4.26 ± 0.17	4.05 ± 0.11	4.17 ± 0.12
Total uMARS score, mean ± SD	3.91 ± 0.17	4.02 ± 0.16	3.78 ± 0.12	3.90 ± 0.12

Legend. SD: Standard Deviation; n: number; uMARS: User Version of the Mobile App Rating Scale.

**Table 4:** Correlations between knowledge score and main study variables.

Variable	Mean ± SD	r	*p*
Total uMARS score	3.91 ± 0.17	0.943	<0.001
Age (years)	40.0 ± 6.9	0.288	0.043
Total work experience (years)	16.0 ± 6.9	0.288	0.043
Experience in current setting (years)	7.0 ± 3.2	0.291	0.041

Legend. SD: Standard Deviation; uMARS: User Version of the Mobile App Rating Scale; r: Pearson’s correlation coefficient; *p*: *p*-value.

## Data Availability

The datasets generated and/or analyzed during the current study are available from the corresponding authors on reasonable request.

## Supplementary Materials

The following supporting information can be downloaded at: https://www.mdpi.com/article/10.3390/jpm16090456/s1. File S1, STROBE Statement—checklist of items that should be included in reports of observational studies [35].

## Appendix A

Italian Society of Nephrology Nurses (SIAN) Board of Directors Group (contributed as authors of the manuscript):

Domenica Gazineo: Italian Society of Nephrology Nurses (SIAN) Board of Directors Group, Via Capotesta 1/30, 07026 Olbia, Italy; Governo Clinico e Qualità, IRCCS Azienda Ospedaliero-Universitaria di Bologna, Bologna, Italy; domenica.gazineo3@unibo.it;

Silvia Cappelletti: Italian Society of Nephrology Nurses (SIAN) Board of Directors Group, Via Capotesta 1/30, 07026 Olbia, Italy; silviacappelletti74@outlook.com;

Alessandro Pizzo: Italian Society of Nephrology Nurses (SIAN) Board of Directors Group, Via Capotesta 1/30, 07026 Olbia, Italy; alessandro.pizzo@fmc-ag.com;

Angela Greco: Italian Society of Nephrology Nurses (SIAN) Board of Directors Group, Via Capotesta 1/30, 07026 Olbia, Italy; angelagreco1967@gmail.com;

Francesco Barci: Italian Society of Nephrology Nurses (SIAN) Board of Directors Group, Via Capotesta 1/30, 07026 Olbia, Italy; francescobarci81@gmail.com;

Addolorata Palmisano: Italian Society of Nephrology Nurses (SIAN) Board of Directors Group, Via Capotesta 1/30, 07026 Olbia, Italy, dorianapalmisano77@gmail.com.
